# Characteristics of Recurrent Visions of the Nonphysical World Among Cognitively Unimpaired Elders of the Ojibwe Tribal Nation

**DOI:** 10.1001/jamanetworkopen.2023.38221

**Published:** 2023-10-18

**Authors:** William G. Mantyh, Adam D. Block, Madelyn R. Castro, Adam Hansen, Matti J. Matheson, Corey Strong, Annamarie Hill, Zuzan Cayci, J. Neil Henderson

**Affiliations:** 1 Department of Neurology, University of Minnesota, Minneapolis; 2 Department of Psychological Sciences, Rice University, Houston, Texas; 3 Bois Forte Health Services, Bois Forte Band of Chippewa, Nett Lake, Minnesota; 4 Memory Keepers Medical Discovery Team, University of Minnesota Duluth, Duluth; 5 Community Outreach, Strategy, External Relations, University of Minnesota Duluth, Duluth; 6 Department of Radiology, University of Minnesota, Minneapolis; 7 Department of Family Medicine and Biobehavioral Health, University of Minnesota Duluth, Duluth

## Abstract

**Importance:**

Visual hallucinations are a core feature of dementia with Lewy bodies and primary psychiatric disease, yet identification of a hallucination vs normal spiritual experience depends on cultural context. Almost no information exists in the medical literature regarding normal spiritual experiences in American Indian participants in the context of a neurocognitive evaluation.

**Objective:**

To assess the characteristics of a normal spiritual experience in an Ojibwe Tribal Nation.

**Design, Setting, and Participants:**

This prospective, cross-sectional study was conducted between August 1, 2021, and August 31, 2022, among an Ojibwe Tribal Nation in northern Minnesota. Participants were evaluated at their tribal nation clinic. Cognitively unimpaired tribal Elders who were enrolled members of the tribal nation and aged 55 years or older were invited to participate via fliers, radio advertisements, and health fair presentations. Thirty-seven tribal Elders volunteered.

**Main Outcomes and Measures:**

Each participant was asked whether they experienced hallucinations or visions of people, animals, or objects that are not part of the physical world. This was an a priori formulated question and part of a comprehensive neurocognitive evaluation consisting of history and physical examination (including cognitive screening with a subspecialty-trained behavioral neurologist); blood tests for metabolic, nutritional, and thyroid conditions; and noncontrast magnetic resonance imaging brain scan. Four patients were excluded from the present analysis due to having mild cognitive impairment or dementia.

**Results:**

Thirty-three cognitively unimpaired tribal Elders (mean [SD] age, 66.0 [7.5] years; 22 women [67%]) were included. Sixteen (48%) answered affirmatively, reporting recurrent visions of the nonphysical world. Generally, these visions were well formed, benevolent in nature, and transient; started in preadolescence; involved spirits or ancestors; and were congruent with cultural and spiritual beliefs of the Ojibwe people. No patients had accompanying dream enactment behavior, dysautonomia, parkinsonism, sleep transition–related hallucinations, or moderate to severe depression to suggest a prodrome of an α-synucleinopathy, hypnopompic or hypnagogic hallucinations, or psychosis.

**Conclusions and Relevance:**

Although based on only 1 Ojibwe Tribal Nation, this study suggests that formed visions of the nonphysical world are common among cognitively healthy Ojibwe individuals and can represent normal spiritual experiences. Clinicians would benefit from careful consideration of cultural or spiritual context to avoid misdiagnosis of neuropsychiatric disease.

## Key Points

**Question:**

What are the characteristics of Ojibwe culture and spirituality in the context of a standard behavioral neurology evaluation?

**Findings:**

In this cross-sectional study partnering with an Ojibwe Tribal Nation in Minnesota, 33 cognitively healthy tribal Elders aged 55 years or older were recruited. Sixteen (48%) reported frequent visions of the nonphysical world that generally involved deceased ancestors or human spirits; these visions were transient, nonthreatening, and carried spiritual significance.

**Meaning:**

This study suggests that, when a diagnosis of a hallucination can disqualify a patient for new Alzheimer disease–modifying therapy, physicians must carefully consider cultural context to avoid misdiagnosis of neuropsychiatric disease.

## Teaser

This cohort study assesses visions of the nonphysical world as part of a normal spiritual experience in the context of a standard behavioral neurology evaluation among Elders in the Ojibwe Tribal Nation.

## Introduction

Hallucinations, or a sensory perception in the absence of a corresponding external stimulus, are considered pathologic if they are not a normal part of the patient’s culture or belief system.^1^ The identification of a hallucination is an integral component of not only primary psychiatric disease but also of neurodegenerative disease. For instance, the presence of early hallucinations with progressive dementia suggests dementia with Lewy bodies and argues against a diagnosis of Alzheimer disease.^2^ The identification of hallucinations also has therapeutic implications not only in the consideration of antipsychotic treatment but also for new Alzheimer disease therapy; hallucinations are possible grounds for exclusion from lecanemab treatment, the first clearly beneficial disease-modifying therapy for Alzheimer disease.^3^

Complicating the interpretation of hallucinations is the patient’s culture or belief system. A person seeing children in the kitchen who have no physical basis would likely be interpreted differently than a monastic individual experiencing a transient vision of a divine being.^1^ The *Diagnostic and Statistical Manual of Mental Disorders* (Fifth Edition) emphasizes that a visual experience without a physical basis is only pathologic if it is not based on the patient’s corresponding culture, religion, or belief system.^1^

Little is known regarding how American Indian culture and spirituality can affect the interpretation of hallucinations in the context of a medical or neuropsychiatric evaluation. The heterogeneity among American Indian tribal nations prevents any broad generalizations. The present study focuses on the Ojibwe (sometimes referred to as *Chippewa*) Tribal Nation in northern Minnesota. The terms *Chippewa* and *Ojibwe* are English and French exonyms for the Anishinaabe people. The term *Chippewa* is used for legal and treaty purposes and is incorporated into the official names for tribal nations of the Anishinaabe people. The term *Ojibwe*, however, is commonly used to describe the ethnolinguistic and ancestral aspect of the Anishinaabe people and is frequently used interchangeably with the term *Anishinaabe*, at least in the United States.^4^ The Ojibwe are the fifth most populous Indigenous people in the US.^5^ Our study seeks to characterize spiritual experiences among Ojibwe Elders that can inform physicians’ diagnosis of a hallucination.

## Methods

This prospective, cross-sectional cognitive aging clinical research study was conducted between August 1, 2021, and August 31, 2022. Each participant (or their legal authorized representative if cognitive loss was to an extent that precluded informed consent) provided written informed consent at the Bois Forte Band of Chippewa (population: 984) Health Services (Nett Lake or Lake Vermilion, Minnesota). Payment of $150 was provided for participation. The Bois Forte Band of Chippewa partnered with the research team, agreed to this work (through a signed, unanimously agreed-on tribal resolution), and agreed to the publication and public dissemination of this article (unanimous agreement at a tribal council meeting). This study was also approved by the University of Minnesota institutional review board. This study follows the Strengthening the Reporting of Observational Studies in Epidemiology (STROBE) reporting guideline.

Using radio interviews, fliers, health fair presentations, and a YouTube video posted to the Ojibwe Tribal Nation social media account, we announced that researchers were hoping to include American Indian participants in cognitive aging clinical research. Eligibility criteria included (1) enrollment in 1 of the 6 Minnesota Chippewa (Ojibwe) Tribal Nations, (2) aged 55 years or older, (3) English as a first language, and (4) absence of an unstable medical condition that requires hospitalization. Normal cognition was defined as having no impairment in instrumental activities of daily living, having not delegated tasks to someone else due to cognitive decline, and scoring 27 or more of a possible score of 30 on the Mini-Mental Status Examination (MMSE) or 36 or more of a possible score of 39 on the Canadian Indigenous Cognitive Assessment (CICA). The CICA includes culturally relevant confrontational naming and memory items and also involves recognition memory tests, which provide greater cultural sensitivity and offer additional differentiation of retrieval vs recognition memory.^6^ Despite the CICA’s development with Ojibwe First Nation individuals in Canada who have a shared entholinguistic history with the Ojibwe Tribal Nations in Minnesota, cultural heterogeneity between regions exists, which led us to also include the MMSE as a cognitive screening tool.^6,7^ Mild cognitive impairment due to Alzheimer disease, dementia with Lewy bodies, and vascular dementia were diagnosed according to previously described criteria.^2,8,9^

Each participant underwent a standard behavioral neurology evaluation with the study’s subspecialty trained behavioral neurologst and principal investigator (W.G.M.), which included history of present illness; 10-point review of systems (including standard behavioral neurology questions about the presence or absence of visual hallucinations [each patient was asked, “Do you have hallucinations or visions of people, animals, or objects that are not part of the physical world?”], other nonvisual hallucinations, delusions, sleep disorders, mood disorders, movement disorders, headache, or seizures); medication list; medical history; family history; social history (including head trauma; occupational history; and alcohol, sedative, recreational, religious, or psychotropic substance review); cognitive screening (MMSE and CICA); and neurologic examination. An informal, qualitative discussion with an Ojibwe Mide practitioner (“medicine man”) was held in which the study team explained the aggregated descriptions of the participants’ visions, including (1) starting in preadolescence or young adulthood, (2) being spiritually meaningful, and (3) involving ancestors or other humans. We enquired as to whether the Ojibwe Mide practitioner considered these a common expression of Ojibwe spirituality.

Participants underwent blood testing for standard reversible dementia markers, including vitamin B_12_ and methylmalonic acid levels; thyroid-stimulating hormone and thyroxine levels; and a comprehensive metabolic panel. A 1.5-T semitruck magnetic resonance imaging (MRI) system was brought to the local clinic, and noncontrast MRI scans were interpreted by a board-certified neuroradiologist (Z.C.) and reviewed by a behavioral neurologist (W.G.M.) to evaluate for any structural brain disease that could explain a participant’s cognitive and neuropsychiatric examination. No statistical tests were performed, as this was a descriptive study.

## Results

Forty-four participants contacted the study team for enrollment. Seven were unable to schedule a visit or were unable to attend their scheduled time. Thirty-seven participants underwent the comprehensive neurocognitive evaluation. These 37 participants were a mean (SD) age of 67.1 (8.1) years, 23 (62%) were female, the mean (SD) years of formal school-based education was 13.1 (1.8) years, the mean (SD) MMSE score was 29.0 (1.8), and the mean (SD) CICA score was 36.9 (1.4). Thirty-three participants had normal cognition; these 33 participants were a mean (SD) age of 66.0 (7.5) years, 22 (67%) were female, the mean (SD) years of formal school-based education was 13.2 (1.5) years, the mean (SD) MMSE score was 29.4 (0.8), and the mean (SD) CICA score was 37.1 (1.2). Four participants had mild cognitive impairment or dementia (2 had mild cognitive impairment [1 due to Alzheimer disease and 1 due to sedating medication use], 1 had vascular dementia, and 1 had dementia with Lewy bodies).

A total of 16 cognitively normal participants (48%) reported well-formed visions of the nonmaterial world that generally involved deceased ancestors or human spiritual beings. These 16 participants were a mean (SD) age of 64.5 (7.7) years, 13 (81%) were female, the mean (SD) years of formal school-based education was 13.0 (1.8) years, the mean (SD) MMSE score was 29.4 (0.8), and the mean (SD) CICA score was 37.1 (1.4). Some of these spiritual visions had unwitnessed interactions with the material world, such as opening doors, making noises on a different floor of the house, or changing the direction a bed was facing. These visions were nonthreatening, transient (generally less than several seconds), present since childhood, and were considered by the participant to be a normal part of their culture’s spiritual experience. We discussed these visions with an Ojibwe Mide practitioner, who assessed them as normal expressions of Ojibwe spirituality. Two of 4 cognitively impaired participants (50%; 1 participant with a diagnosis of dementia with Lewy bodies and 1 with a diagnosis of vascular dementia) had similar spiritual visual experiences. Other additional hallmark features of their respective clinical diagnoses were present to the extent that their diagnoses were not based on the presence or misattribution of their spiritual experiences as hallucinations.

None of the participants with normal cognition had dream enactment behavior, parkinsonism, sleep-related hallucinations, dysautonomia, fluctuations in mental status, metabolic derangements beyond mild hyperglycemia or mild hyponatremia, current moderate to severe depression, or psychedelic or recreational substance use. None of the participants had any evidence of disorganized thoughts, delusional thinking, or prior or current severe psychiatric illness or psychiatric hospitalization. Of the 24 participants with normal cognition who underwent MRI (9 did not participate due to scheduling conflicts, claustrophobia, or MRI-incompatible metallic implants or shrapnel), none had any structural lesions or abnormalities on neuroimaging scans beyond mild small vessel disease of the subcortical white matter. There were no interpretation disputes between the interpreting neuroradiologist (Z.C.) and the behavioral neurologist (W.G.M.).

## Discussion

Visions of the nonmaterial world are common among cognitively healthy members of an Ojibwe Tribal Nation and reflect normal spiritual experiences. These visions were present since childhood, nonthreatening, transient, generally related to ancestors or human spirits, and carried spiritual significance. Unlike psychiatric features commonly encountered among individuals with neurodegenerative disease, none of our participants (including those with cognitive impairment) had visions accompanied by paranoid, persecutory, grandiose, or personal misidentification thought content.^10^ Apart from visual spiritual experiences that could be misinterpreted as hallucinations, none of the participants with normal cognition had any core features of dementia with Lewy bodies^2^ or psychosis.^1^ Although the religious beliefs of the Ojibwe are heterogeneous and vary from person to person, a common thread is that animals, plants, and nature are spiritually inhabited and sacred.^11^ This harmony of spiritual life within the environment is in keeping with the regularly occurring, spiritually meaningful experiences of the participants in this study.

### Limitations and Strengths

This study has some limitations. One is generalizability because this study was conducted in 1 Ojibwe Tribal Nation. There are 574 federally recognized tribal nations in the United States with enormous linguistic, cultural, and spiritual heterogeneity.^12^ Given that other Indigenous peoples in the US and North America have similar epistemologies linking human experience with the spiritual and natural environment,^13^ future work is warranted to determine whether the findings herein extend more generally to other Indigenous groups. The second limitation is selection bias; data herein stem from a cognitive aging clinical research study, which may result in recruitment of an unrepresentative sample. Religious psychedelic substance use was not a confounding factor or limitation; as opposed to some American Inidan spiritual practices most prevalent in the southwest US, peyote and religious psychedelics are not used among the Ojibwe people and were not reported by any participant.^14^

The study’s strength is that it was performed at the Ojibwe Tribal Nation clinic with daily in-person assistance of tribal health staff, which created a nonintimidating environment for participants to openly share their spiritual beliefs and helped to promote the recruitment of underrepresented Indigenous people who otherwise might not be part of clinical research.

## Conclusions

Today’s environment of infrequent or insufficiently short cognitive evalutions,^15,16^ a mean 16-minute face-to-face visit with a physician,^17^ and increasing use of previsit symptom checklists^18^ increase the risk of falsely attributing a spiritual experience to a hallucination. Consideration of a patient’s cultural background and belief system can help avert erroneous disqualification for disease-modifying therapy (such as lecanemab),^3^ exclusion from clinical trials,^19^ and all the negative ramifications associated with a misdiagnosis of psychiatric disease. Novel biomarkers for dementia with Lewy bodies^20,21^ and other neurodegenerative diseases^22,23^ represent promising additional safeguards to prevent misdiagnosis among participants with normal cultural spiritual experiences.

## Notes

Supplement.
Data Sharing Statement

## References

[1] American Psychiatric Association. Schizophrenia spectrum and other psychotic disorders. In: Diagnostic and Statistical Manual of Mental Disorders. 5th ed. American Psychiatric Association; 2013.

[2] McKeith IG, Boeve BF, Dickson DW, . Diagnosis and management of dementia with Lewy bodies: fourth consensus report of the DLB Consortium. Neurology. 2017;89(1):88-100. doi:10.1212/WNL.0000000000004058 28592453 PMC5496518

[3] Cummings J, Apostolova L, Rabinovici GD, . Lecanemab: appropriate use recommendations. J Prev Alzheimers Dis. 2023;10(3):362-377. doi:10.14283/jpad.2023.30 37357276 PMC10313141

[4] National Endowment for the Humanities. Anishinabe/Ojibwe/Chippewa: culture of an Indian Nation. Accessed August 28, 2023. https://edsitement.neh.gov/lesson-plans/anishinabeojibwechippewa-culture-indian-nation

[5] United States Census Bureau. American Community Survey: American Indian and Alaska Native alone for selected tribal groupings. 2021. Accessed March 20, 2023. https://data.census.gov/table?q=American+Indian+and+Alaska+Native&tid=ACSDT1Y2021.B02014

[6] Jacklin K, Pitawanakwat K, Blind M, . Developing the Canadian Indigenous Cognitive Assessment for use with indigenous older Anishinaabe adults in Ontario, Canada. Innov Aging. 2020;4(4):igaa038. doi:10.1093/geroni/igaa038 33072890 PMC7545789

[7] Folstein MF, Folstein SE, McHugh PR. “Mini-mental state”: a practical method for grading the cognitive state of patients for the clinician. J Psychiatr Res. 1975;12(3):189-198. doi:10.1016/0022-3956(75)90026-6 1202204

[8] Albert MS, DeKosky ST, Dickson D, . The diagnosis of mild cognitive impairment due to Alzheimer’s disease: recommendations from the National Institute on Aging–Alzheimer’s Association workgroups on diagnostic guidelines for Alzheimer’s disease. Alzheimers Dement. 2011;7(3):270-279. doi:10.1016/j.jalz.2011.03.008 21514249 PMC3312027

[9] Román GC, Tatemichi TK, Erkinjuntti T, . Vascular dementia: diagnostic criteria for research studies: report of the NINDS-AIREN International Workshop. Neurology. 1993;43(2):250-260. doi:10.1212/WNL.43.2.250 8094895

[10] Naasan G, Shdo SM, Rodriguez EM, . Psychosis in neurodegenerative disease: differential patterns of hallucination and delusion symptoms. Brain. 2021;144(3):999-1012. doi:10.1093/brain/awaa413 33501939 PMC8041322

[11] Ondich J. *World Religions: The Spirit Searching*. Creative Commons; 2021. Accessed August 28, 2023. https://mlpp.pressbooks.pub/worldreligionsthespiritsearching/

[12] Federal Register. Indian entities recognized by and eligible to receive services from the United States Bureau of Indian Affairs. January 29, 2021. Accessed May 2, 2023. https://www.federalregister.gov/documents/2021/01/29/2021-01606/indian-entities-recognized-by-and-eligible-to-receive-services-from-the-united-states-bureau-of

[13] Forbes JD. Indigenous Americans: spirituality and ecos. *Daedalus*. 2001;130(4):283-300. Accessed September 14, 2023. https://www.jstor.org/stable/20027728

[14] Gordon BA, Blazey TM, Su Y, . Spatial patterns of neuroimaging biomarker change in individuals from families with autosomal dominant Alzheimer’s disease: a longitudinal study. Lancet Neurol. 2018;17(3):241-250. doi:10.1016/S1474-4422(18)30028-0 29397305 PMC5816717

[15] Kotagal V, Langa KM, Plassman BL, . Factors associated with cognitive evaluations in the United States. Neurology. 2015;84(1):64-71. doi:10.1212/WNL.0000000000001096 25428689 PMC4336093

[16] Hinton L, Franz CE, Reddy G, Flores Y, Kravitz RL, Barker JC. Practice constraints, behavioral problems, and dementia care: primary care physicians’ perspectives. J Gen Intern Med. 2007;22(11):1487-1492. doi:10.1007/s11606-007-0317-y 17823840 PMC2219799

[17] Young RA, Burge SK, Kumar KA, Wilson JM, Ortiz DF. A time-motion study of primary care physicians’ work in the electronic health record era. Fam Med. 2018;50(2):91-99. doi:10.22454/FamMed.2018.184803 29432623

[18] Sinsky CA, Sinsky TA, Rajcevich E. Putting pre-visit planning into practice. Fam Pract Manag. 2015;22(6):34-38. 26761083

[19] Humphreys K, Blodgett JC, Roberts LW. The exclusion of people with psychiatric disorders from medical research. J Psychiatr Res. 2015;70:28-32. doi:10.1016/j.jpsychires.2015.08.005 26424420

[20] Russo MJ, Orru CD, Concha-Marambio L, . High diagnostic performance of independent alpha-synuclein seed amplification assays for detection of early Parkinson’s disease. Acta Neuropathol Commun. 2021;9(1):1-13. 34742348 doi:10.1186/s40478-021-01282-8 34742348 PMC8572469

[21] Bellomo G, De Luca CMG, Paoletti FP, Gaetani L, Moda F, Parnetti L. α-Synuclein seed amplification assays for diagnosing synucleinopathies: the way forward. Neurology. 2022;99(5):195-205. doi:10.1212/WNL.0000000000200878 35914941

[22] Mattsson N, Andreasson U, Zetterberg H, Blennow K; Alzheimer’s Disease Neuroimaging Initiative. Association of plasma neurofilament light with neurodegeneration in patients with Alzheimer disease. JAMA Neurol. 2017;74(5):557-566. doi:10.1001/jamaneurol.2016.6117 28346578 PMC5822204

[23] Karikari TK, Pascoal TA, Ashton NJ, . Blood phosphorylated tau 181 as a biomarker for Alzheimer’s disease: a diagnostic performance and prediction modelling study using data from four prospective cohorts. Lancet Neurol. 2020;19(5):422-433. doi:10.1016/S1474-4422(20)30071-5 32333900

