# Supplementary material for: Characteristics of Recurrent Visions of the Nonphysical World Among Cognitively Unimpaired Elders of the Ojibwe Tribal Nation
Source: JAMA Netw Open. 2023 Oct 18;6(10):e2338221. doi: 10.1001/jamanetworkopen.2023.38221 (PMC10585403; doi:10.1001/jamanetworkopen.2023.38221)
Supplement: Supplement. — Data Sharing Statement [file jamanetwopen-e2338221-s001.pdf]

Data Sharing Statement

Mantyh. Characteristics of Recurrent Visions of the Nonphysical World Among Cognitively Unimpaired Elders of the Ojibwe Tribal Nation. *JAMA Netw Open*. Published October 18, 2023. doi:10.1001/jamanetworkopen.2023.38221

Data
Data available: No

Additional Information
Explanation for why data not available: Data sharing for individual participants may be possible, however current agreement between investigative team and Tribal Nation is that no individual level data will be shared, only aggregate data.
